# Spiritual wellbeing in psychedelic-assisted therapy with palliative care populations: An analysis of outcome measures

**DOI:** 10.1017/S1478951526101801

**Published:** 2026-02-27

**Authors:** Stephen Lewis

**Affiliations:** 1 Spiritual Care Services, University of Maryland, Baltimore, MD, USA; 2 Spiritual Care Services, University of California, San Diego , CA, USA; 3 Emory Center for Psychedelics and Spirituality, Emory University, Atlanta, GA, USA

**Keywords:** psychedelic-assisted therapy, spiritual well-being, outcome measures, instruments, palliative care

## Abstract

**Objectives:**

People with serious illnesses often experience spiritual and emotional pain, manifesting in conditions such as depression, anxiety, and demoralization. Emerging research in psychedelic-assisted therapy has shown efficacy in treating these conditions. Despite evidence that psychedelics frequently occasion mystical/spiritual experiences in participants, there has been little research on support for spiritual, existential, religious, and theological needs, including the use of chaplains on therapeutic teams. Spiritual wellbeing outcomes have been inconsistently used and reported on in current psychedelics studies. The aims of this article are to identify and review patient-centered outcome measures focused on spiritual wellbeing for use in psychedelic research.

**Methods:**

A literature review of instruments was conducted, with 286 articles included, identifying spiritual wellbeing measures within the palliative care population.

**Results:**

Three measures were selected for inclusion: Functional Assessment of Chronic Illness Therapy-Spiritual Well-Being 12- Item Scale (FACIT-Sp-12), European Organization for Research and Treatment of Cancer Quality of Life Spiritual Well-being Questionnaire (EORTC QLQ-SWB-32), and the National Institutes of Health Healing Experience of All Life Stressors (NIH-HEALS). Instrument development, psychometric properties, and use in research for each are discussed.

**Significance of Results:**

Suitability in the context of psychedelic-assisted therapy with the palliative care population includes strong reliability and validity, and they should be accessible to people with various spiritual traditions, practices, and sources of connection. They should be patient-centered in their development, involve multiple stakeholders, and be appropriate for use with palliative care populations. According to these criteria and its orientation toward identifying spiritual change in the context of serious illness, the NIH-HEALS is recommended for wider use in psychedelic-assisted therapy.

## Introduction

In addition to physical symptoms, people diagnosed with serious illnesses experience emotional and spiritual pain which frequently presents in the form of mental health diagnoses such as depression, anxiety, post-traumatic stress disorder (PTSD), and demoralization (Miller et al. 2023). The National Consensus Project Clinical Practice Guidelines for Quality Palliative Care (NCP Guidelines) include attention to psychological, social, and spiritual needs of patients and caregivers (Ferrell et al. 2018). Palliative Care supports patients through the use of interdisciplinary teams including physicians, advanced practice professionals, nurses, social workers, and chaplains (Ferrell et al. 2018).

There has been substantial growth in addressing some of the mental health concerns noted above through the use of psychedelic-assisted therapies (PAT), including substances such as psilocybin (“magic mushrooms”), lysergic acid diethylamide (LSD), ketamine, *N,N*-dimethyltryptamine (DMT), mescaline, ibogaine, and 3,4-methylenedioxymethamphetamine (MDMA) (Aday et al. 2020). Studies with psilocybin, in particular, have produced rapid and significant improvements in depression and anxiety for people diagnosed with life-threatening cancer (Griffiths et al. 2016; Ross et al. 2016). Increasing attention has been given to applying PAT specifically within the palliative care population (Ross et al. 2022). For example, 3 psilocybin trials are currently underway within the hospice and palliative care population (Beaussant 2023; Zarrabi 2023; Grob 2025), each of which focus on demoralization, a condition characterized by despair, hopelessness, and helplessness (Fava et al. 2023).

Among the interesting aspects of PAT is the consistent observation of mystical-type experiences among participants which are deeply meaningful (Griffiths et al. 2006, 2025). Some have argued that these subjective effects are necessary for enduring benefit (Yaden and Griffiths 2021). One systematic review suggests that mystical experiences in PAT may mediate clinical improvements and therapeutic efficacy (Ko et al. 2022). This has led to calls for researchers and clinicians to specifically attend to spiritual, existential, religious, and theological (SERT) components in PAT, including the use of chaplains in therapeutic treatment protocols (Palitsky et al. 2023; Griffiths et al. 2025).

In palliative care, spiritual assessment is an important function of chaplains, to gather spiritual history, beliefs, importance of practices, and interpretive frameworks, and to discover patients’ spiritual needs to include in plans of care (McCann Klug 2023). Several spiritual assessment models have been used, with a variety of emphasis on categories of meaning, spiritual needs in illness, and implementation (Galchutt 2024). However, there are no published spiritual assessment models specific to PAT, which represents a gap in understanding the spiritual effects in PAT, and potentially limits the benefits gained. Additionally, though chaplain contributions to PAT have been described (Palitsky et al. 2023; Peacock et al. 2024; Pasricha et al. 2025), no published studies to date have examined quantitative treatment effects (if any) of chaplains as members of therapeutic teams in PAT. With the above in mind, this article aims to identify and describe spiritual wellbeing outcome measures that could be used in PAT studies that include chaplain-led spiritual assessments, and to assess their psychometric properties.

## Literature review

While SERT dynamics have gained the interest of researchers, there has not been substantial agreement as to which outcome measures to use in PAT (Baker et al. 2023). Further, the reporting of protocols, measures, and findings in PAT have been heterogeneous, leading to confusion in this regard (Pronovost-Morgan et al. 2025). The most commonly used measures, such as the Mystical Experience Questionnaire (MEQ), are retrospective in nature, only describing subjective effects after dosing sessions in PAT, and may not indicate whether these effects result in changes to dimensions of wellbeing (Barrett et al. 2015). Some spiritual wellbeing measures have been implemented, but due to heterogeneous reporting, it is unclear how they have been used (Baker et al. 2023).

In evaluating outcome measures, it should be noted that most spiritual wellbeing instruments in published PAT studies were not developed with PAT populations in mind. While these instruments may have been validated in multiple populations, including people with serious illness, it is important that they be appropriate to the palliative care population and consider the potential areas of vulnerability (Addington-Hall 2002). For example, Davis et al. (2025) describe a palliative care psilocybin study in which some participants experienced disease-related pain exacerbations during their dosing sessions, and the therapeutic team had to decide whether to administer their usual doses of opioid medications at the risk of blunting psilocybin effects. In this scenario, outcome measures used at the end of the dosing sessions could potentially be compromised due to the continued influence of the opioids. Ethical vigilance in research requires that the use of instruments comes second to the health of participants.

Ethical considerations must also be attended to in the use of outcome measures in palliative care PAT studies. Lengthy self-report questionnaires following dosing sessions that are typically 6–8 hours in duration may be burdensome for people with serious illness, especially as they may be more vulnerable to physical and emotional fatigue. Selection of outcome measures must also be done with attention to validity issues (DeVon et al. 2007; Granda-Cameron et al. 2008), which is particularly the case with variable definitions of “spiritual” or “mystical” (Baker et al. 2023) in PAT studies. Informed consent is a critical issue in PAT, as participants in dosing sessions are highly suggestible and vulnerable to coercive behavior on the part of the therapeutic team, which may include the use of outcome measures, and may require extra steps of caution (Carpenter et al. 2021; Marks et al. 2024). Similarly, the importance of the therapeutic alliance between PAT participants and treatment teams has been suggested in some studies (Murphy et al. 2021), which may raise some concerns related to participants feeling pressured to report more positive effects on certain outcome measures. Finally, ethical considerations should be given to a wide variety of stakeholders in PAT research, which, in addition to participants and their caregivers, concerns therapeutic team members, researchers, insurers, and regulatory agencies (Marks and Cohen 2023).

Psychometric properties must also be considered in palliative care PAT outcome measures. Using reliable instruments that demonstrate consistency in measurement and over spans of time is critically important. Test–retest reliability, for example, can be a concern in palliative care populations due to change in illness trajectories that may affect item responses over time. The validity of measures is also essential, as researchers need to be fully confident that they are actually measuring what they set out to (DeVon et al. 2007). For example, criterion validity is important in this particular context as terms such as “spiritual” have a diversity of definitions (Baker et al. 2023) and cultural presuppositions, and instrument items must be broad enough to take in multiple religious and cultural understandings.

**Figure 1.**
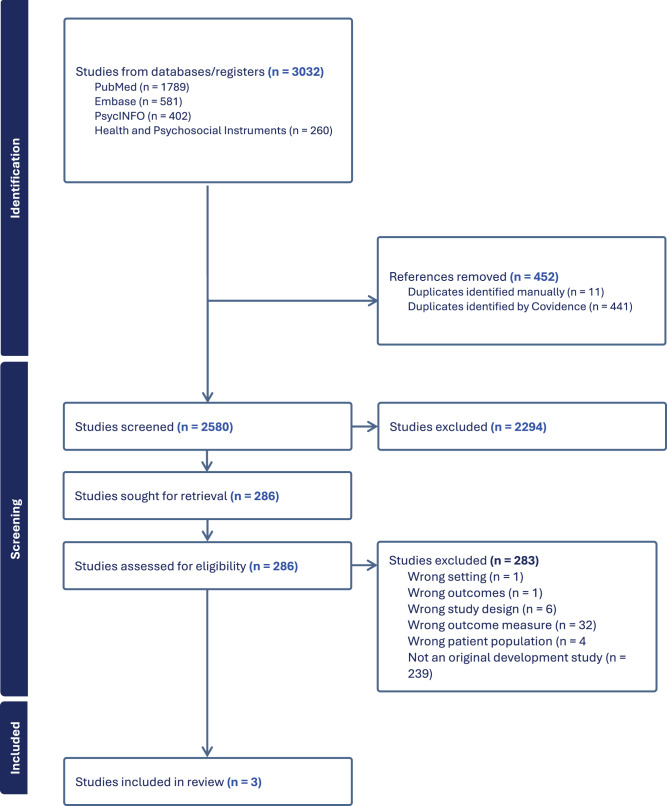
Preferred Reporting Items for Systematic Reviews and Meta-Analyses (PRISMA) flow diagram for the literature review. Embase, PubMed, PsycInfo, Health and Psychosocial Instruments (HAPI).

For this literature review, a search of Embase, PubMed, PsycInfo, and Health and Psychosocial Instruments (HAPI) was conducted in June 2025. The search terms used were (“spiritual wellbeing” OR “spiritual well-being”) AND (“measure*” OR “instrument” OR “inventory” OR “tool” OR “questionnaire” OR “survey” OR “test”) AND (“palliative” OR “serious illness”). A total of 3,032 results were returned and screened in Covidence. See Fig. 1 for PRISMA diagram. After removal of duplicates and an initial screening for relevance, 286 results were manually screened according to review criteria. Articles excluded from this review included non-English language studies, non-adult population studies, related to caregivers or clinicians, non-palliative/serious illness populations, non-full text articles, and those without a spiritual wellbeing outcome measure identified.

Included articles were reviewed to identify aspects of spiritual wellbeing that were assessed, how frequently specific spiritual wellbeing outcome measures were used, and how broadly the measures were used within palliative care populations. Observations were also made regarding the use of measures in different languages, countries, and cultures. Based on these criteria, 3 instruments were selected and will be reviewed in the sections below.

## Findings

### Functional Assessment of Chronic Illness Therapy – Spiritual Well-Being 12-Item Scale (FACIT-Sp-12)

The FACIT-Sp-12 is one of the most widely used measures of spiritual wellbeing. This instrument is an inclusive measure of spirituality in research with people with chronic and/or life-threatening illness (Peterman et al. 2002). It is part of the larger Functional Assessment of Cancer Therapy-General (FACT-G), a health-related quality of life measure. It was developed from interview data from 135 cancer patients and 15 oncology specialists related to aspects of spirituality and/or faith in quality of life. In the original FACT-G development, items related to spirituality and faith that affected quality of life were dropped due to validation concerns. However, the interviews related to this development emphasized the significance of spirituality and faith in illness with this population, so this 12-tem scale was developed through original FACT-G interviews and subsequent interviews with over 200 patients as well as interviews with hospital chaplains.

Two validation studies were done to assess the FACIT-Sp-12. The first was done with 1,617 subjects (83.1% with cancer) across 4 sites in the mainland USA and 3 sites in Puerto Rico to establish the factor structure. Factor analysis was done with varimax rotation to test unidimensionality. Two factors/subscales were identified: Meaning/Peace and Faith, with correlation of 0.54 (*P* = 0.0001) between the subscales. Reliability was evaluated via internal consistency tests with very good alpha coefficients (Cronbach’s α = 0.81–0.88) (see Table 1). Validity was done through Spearman correlation analysis with the original FACT-G and its subscales (correlation 0.31–0.62 for Meaning/Peace, 0.09–0.35 for Faith, 0.25–0.58 for FACIT-Sp-12 total), Profile of Mood States – Short Form (POMS-SF) (negative correlations with tension, depression, anxiety, fatigue, and confusion subscales of −0.39 to −0.60 for Meaning/Peace, −0.21 to − 0.30 for Faith, −0.36 to −0.54 for FACIT-Sp-12 total), and Marlowe-Crowne Social Desirability Scale (MCSDS) (correlation of 0.22, 0.26, and 0.27 for Meaning/Peace, Faith, FACIT-Sp-12 total, respectively). The second validation study was done with 131 participants to further validate the measure in comparison to 5 other existing measures in 6 dimensions of religion and spirituality: organizational religious activity (ORA) and non-organizational religious activity (NORA), spiritual beliefs and religious social support, coherence, and intrinsic religiosity. For reliability, the mean scores on the FACIT-Sp-12 full scale and subscales were comparable to those in the first study, as were internal consistency estimates for the FACIT-Sp-12, Meaning/Peace, and Faith (Cronbach’s α = 0.86, 0.81, and 0.86, respectively). For convergent validity, the total FACIT-Sp-12 was moderately correlated with all of the other measures (*rs* = 0.31 with NORA to 0.48 to total Spiritual Beliefs Inventory (SBI) score, *P’s* < 0.0005).

**Table 1.** Table of Spiritual Wellbeing Measures

Instrument	Designed to measure	Item development	Validity	Reliability	Populations in which tools was used
Functional Assessment of Chronic Illness Therapy – Spiritual Well-Being 12 Item Scale (FACIT-Sp-12) (Peterman et al. 2002)	Inclusive measure of spirituality in research with people with chronic and/or life-threatening illness.	Part of the larger Functional Assessment of Cancer Therapy-General (FACT-G), a health-related quality of life measure. Developed from interviews with cancer patients and hospital chaplains related to aspects of spirituality and/or faith in quality of life.	Spearman correlations between FACIT-Sp-12 and FACT-G quality of life, 0.31−0.62, 0.09−0.35, 0.25−0.58 for Meaning/Peace, Faith, and FACIT-Sp-12 total, respectively; Inverse correlation to Profile of Mood States – Short Form (POMS-SF), −0.39 to −0.60, −0.21 to −0.30, −0.36 to −0.54 for Meaning/Peace, Faith, and FACIT-Sp-12 total, respectively; and Marlowe-Crowne Social Desirability Scale (MCSDS), 0.22, 0.26, and 0.27 for Meaning/Peace, Faith, FACIT-Sp-12 total, respectively.	Internal consistency, Cronbach’s α = 0.81−0.88.	Developed for use with adult cancer patients. Used widely in other serious illness populations and PAT.
European Organization for Research and Treatment of Cancer Quality of Life Questionnaire-Spiritual Well-being (EORTC QLQ-SWB32) (Vivat et al. 2017)	Stand-alone measure of spiritual well-being for palliative cancer patients. Intentionally developed for use in multiple languages and cultures.	Developed from EORTC QLG Module Development Guidelines (Wheelwright et al. 2021) with stakeholder interviews in 7 countries. Validation study with 451 palliative patients in 14 countries.	Convergent and divergent validity through comparison of EX to EORTC QLQ-PAL global quality of life (G-QL) (Spearman’s rho 0.5000−0.514, *P* < 0.0001) and Emotional Functioning (EF) scales (0.409 to 0.421, *P* < .0001).	Internal reliability, Cronbach’s α = 0.68−0.84. Test–retest reliability, Wilcoxon signed-rank tests showed median score on relationship to self slightly higher at retest (*Z* = − 1.249, − 2.75, −1.784, −1.181).	Developed for palliative care patients with cancer, but has found use with other palliative and chronic disease populations. No published PAT studies to date.
National Institutes of Health Healing Experience of All Life Stressors (NIH-HEALS) (Sloan et al. 2017; Ameli et al. 2018)	Designed to measure positive psychological, social, and spiritual change in the context of serious illness.	Originally a 48-item questionnaire developed from literature review, qualitative data review, and expert panel input. Pilot study done (Sloan et al. 2017), with later validation study (Ameli et al. 2018).	Correlation studies with FACIT-Sp-12 Meaning, Peace, and Faith factor scores (*r*_s_ = 0.62, *P* < 0.0001; *r*_s_ = 0.60, *P* < 0.0001; and *r*_s_ = 0.60, *P* < 0.000, respectively), and Self-Integration Scale (SIS) Codependent factor (inverse correlation) (*r*_s_ = −0.34, *P* < 0.0001).	Internal consistency, Cronbach’s α = 0.89, split-half reliability = 0.95.	Developed for use with patients with severe and/or life threatening disease. Has been used in PAT studies.

The FACIT-Sp-12 has been translated and validated in 39 languages to date (Best et al. 2024). This measure has been used frequently in PAT studies. A recent review of spirituality measures in psilocybin studies with serious illness populations found that it was the most widely used instrument in included investigations (Baker et al. 2023). For example, one study of people with life-threatening cancer and anxiety and/or depression used the FACIT-Sp-12 along other measures (Ross et al. 2016), as did a long-term follow-up 4.5 years after that study (Agin-Liebes et al. 2020). In both cases, spiritual wellbeing and faith domains showed significant improvement with the psilocybin intervention from baseline to follow-up periods of 6.5 months, 3.2 years, and 4.5 years.

The FACIT-Sp-12 has several strengths. In addition to the reliability and validity described above, this instrument examines spirituality rather than specific beliefs or practices, which may make it more compatible with various religious traditions and cultures. Also, given its roots in the FACT-G measure and subscales, this instrument was made with populations with serious illness in mind. Furthermore, the development of the measure clearly incorporated the spiritual values of patients, as their interview responses contributed to the item selection. Other stakeholder clinicians’ involvement signals a thoughtful multi-faceted development process. In terms of limitations, the primary question regarding the FACIT-Sp-12 has to do with follow-up validity studies, and mixed results related to whether it should be comprised of the 2 subscales described above of meaning/peace and faith, or if a 3-factor model would be better, using meaning, peace, and faith (Peterman et al. 2014).

### European Organization for Research and Treatment of Cancer Quality of Life Questionnaire-Spiritual Well-Being (EORTC QLQ-SWB32)

The European Organization for Research and Treatment of Cancer (EORTC) acknowledged that while existing spiritual wellbeing measures may have been translated and validated into multiple languages, none had been developed cross-culturally to address concepts of spirituality and culture that may exist in some cultures, but not others, which led to the development of the EORTC QLQ-SWB36, the original, 36-item spiritual wellbeing measure (Vivat et al. 2013). It was developed using guidelines from the EORTC Quality of Life Group (QLG) (Wheelwright et al. 2021) in 4 stages, with stakeholders in 6 European countries and Japan. A literature review identified relevant issues using a working definition of spirituality, attempting to identify how respondents’ beliefs function in their daily lives. Dimensions of personal relationships with self and others, existential issues, and religious/spiritual beliefs and practices as their dimensions of concern were developed. Interview feedback with patients with cancer receiving palliative care and palliative care clinicians, including at least 1 religious professional from each country, was sought to identify issues and items for the questionnaire. The measure was piloted with 113 people.

A validation study was then conducted involving 451 palliative cancer patients from 14 countries and from different religious backgrounds. The instrument used 5 subscales: Relationships with others (RO), Relationship with Self (RS), Existential (EX), Relationship with Someone or Something Greater (RSG), and Change (CH). Rasch analysis for model fit was done, which identified 4 items for deletion. Internal reliability of the scales was good (Cronbach’s α = 0.68 to 0.84) (see Table 1). Construct validity showed that no significant mean score differences were observed by religious belief or gender overall, but women had significantly higher mean scores on the RSG scale. Correlation analysis was done to assess convergent and divergent validity, comparing this measure’s subscales to the EORTC’s PAL global quality of life (PAL G-QL) and emotional functioning (EF) items. The EX subscale was moderately to strongly correlated with G-QL (Spearman’s rho 0.5000–0.514, *P* < 0.0001) and moderately with EF (0.409 to 0.421, *P* < 0.0001). The RS scale moderately correlated with the EF scale (0.440 to 0.427, *P* < 0.0001), and weakly with G-QL. Test–retest reliability was done with 49 patients in 9 countries, and only the RS scale was significantly different (higher on retest) between administrations.

The EORTC QLQ-SWB32 has been validated in 27 languages. The majority of published articles to date are validation studies of new language versions of the instrument. A recent example of a validation of the measure in Finnish examines correlations of spiritual wellbeing and quality of life in both cancer and non-cancer palliative care patients (Goyarrola et al. 2023). The study reports forward and backward translation from English to Finnish. In addition to face, content, and construct validity, good reliability was reported. Quality of life questionnaires were given to 74 cancer and 72 non-cancer patients. RS, RSG, EX, RG, and global spiritual wellbeing were significantly higher in the non-cancer group compared to the cancer group. Other examples of translation and validation studies include a Chinese version (Feng et al. 2025) and an adaptability study of the Greek version in Cyprus (Kyranou and Nicolaou 2021).

No published PAT studies to date have used the EORTC QLQ-SWB32, so for the purposes of this article, only inferences to palliative care populations can be made. The intentionality of the instrument’s development with multiple countries, languages, and cultures in mind make it appealing for use in a wide range of contexts. The applicability of concepts gleaned from interviews across different countries and languages, with patients, clinicians, and spiritual practitioners strongly suggests that several stakeholder groups were involved in development of the measure. While the development and validation in various contexts may be intriguing to researchers looking for culturally appropriate outcome measures, few non-validation studies using the instrument have been published, which largely leaves its utility to speculation at this time.

### National Institutes of Health Healing Experience of All Life Stressors (NIH-HEALS)

This instrument grew out of observations that some patients, when encountering a life-threatening illness, experience increases in positive change during their diagnosis and treatment of illness, and take place even when poor prognosis and physical outcomes are present (Holder et al. 2015, 2015; Sloan et al. 2017). Despite the existence of a number of instruments to measure quality of life, symptoms, psychosocial, and spiritual dimensions of serious illness, none focused on aspects of “psycho-social-spiritual healing,” which led to the original development of a predecessor version of NIH-HEALS (Sloan et al. 2017). A literature review was done to develop concepts, definitions, and constructs related to healing, and categorized them around themes. For face and content validity, palliative care experts including physicians, nurses, social workers, chaplains, and a scientist with expertise in instrument development reviewed items.

A pilot study with 100 US patients with chronic or life-limiting disease completed a survey, and factor analysis was done to formulate a 48-item questionnaire. A follow-up study with 200 patients reported that the number of items was reduced to 42 items following cognitive interviewing, and later to 35 items through factor analysis and validation, with the items organized around 3 factors: Connection (religious, spiritual, and interpersonal), Reflection & Introspection, and Trust & Acceptance (Ameli et al. 2018). Strong internal consistency and reliability were reported (Cronbach’s α = 0.89, split-half reliability = 0.95) (see Table 1). Strong convergent validity was demonstrated, with correlation studies against FACIT-Sp-12 Meaning, Peace, and Faith factor scores (*r*_s_ = 0.62, *P* < 0.0001; *r*_s_ = 0.60, *P* < 0.0001; and *r*_s_ = 0.60, *P* < 0.000, respectively) and Self-Integration Scale (SIS) S (*r*_s_ = 0.64, *P* < 0.0001). Divergent validity was done with the SIS Codependent factor, and was found to have inverse correlations (*r*_s_ = − 0.34, *P* < 0.0001). Confirmatory factor analysis indicated significant relationships between NIH-HEALS items and corresponding factors.

As a still relatively new measure, NIH-HEALS has only been used in a few cases by investigators. However, pertinent to PAT, it was used in an open-label psilocybin study with 30 cancer patients in the setting of individual and group therapy, with the 3 domains of spiritual wellbeing from NIH-HEALS as the primary outcome variables (Shnayder et al. 2023). The results of this study included improvements across all 3 domains, measured at baseline (prior to dosing) and at 1, 3, and 8 weeks post-dosing. The Connection factor scores increased by 12.7% by week 8 (*P* = 0.003), Reflection & Introspection scores increased by 7.7% by week 8 (*P* < 0.001), and scores on the Trust & Acceptance increased by 22.4% by week 8 (*P* < 0.001). Another study involving ketamine-assisted psychotherapy with people with problematic substance use also used NIH-HEALS as an outcome measure (Whinkin et al. 2023). Data gathered through retrospective chart review showed improvements in depression and anxiety after at least 1 ketamine-assisted psychotherapy session. There was insufficient evidence for statistically significant improvements in NIH-HEALS scores; however, the authors note that outcome measures including NIH-HEALS are routinely collected at 90-day intervals following establishment of treatment, but that the responses to these instruments vary widely in their proximity to ketamine treatments, which makes comparisons difficult.

Understanding the validity and reliability of NIH-HEALS as a research instrument will benefit from increased use and reporting. However, it has multiple areas of strength. For example, it was developed with multiple patient-centered outcomes concerns in mind, especially the inclusion of multiple stakeholders in the development: patients, interprofessional clinicians, and even the institutional backing of the National Institutes of Health. Another distinguishing feature when compared to other spiritual wellbeing measures is that it focuses on concepts of healing, growth, and change, i.e. “positive” outcomes, rather than measures that focus on spiritual “distress” as a starting point. The 3-factor model of NIH-HEALS appears well-designed to help characterize spiritual wellbeing for people of a variety of spiritual and religious traditions.

## Discussion

To date, PAT studies with palliative care populations have primarily focused on mental health outcomes such as depression, demoralization, or anxiety, with spiritual wellbeing as either a secondary outcome or unconsidered. Furthermore, the well-documented mystical and spiritual experiences that study participants describe are frequently either not attended to in research protocols or are under-supported by virtue of the fact that PAT therapeutic support team members may have little training in SERT components (Palitsky et al. 2023). The primary aim of this article is to identify and review spiritual wellbeing outcome measures for use in PAT studies that do address SERT issues and support them through chaplain-led spiritual assessments. Each of the 3 outcome measures reviewed above addresses constructs of spiritual wellbeing and measures them as indicators of quality of life.

The extensive use of the FACIT-Sp-12 in research settings has provided ample evidence of strong reliability in English (Cronbach’s α = 0.81–0.88) and a large number of other language translations. This consistency over time and in a variety of use cases adds credibility to the value of the measures. The NIH-HEALS also demonstrates strong reliability (Cronbach’s α = 0.89–0.95), as does the EORTC QLQ-SW32 to a lesser degree (Cronbach’s α = 0.68–0.84). As newer instruments, they will benefit from further use in palliative care research settings. In terms of validation, each of the 3 measures demonstrates acceptable to strong correlation to comparison scales. However, it is notable that items in EORTC QLQ-SWB32 were only compared to the EORTC QLQ-C15-PAL, a related quality of life questionnaire for palliative care patients, rather than to other existing, related measures. (convergent and divergent validity of EORTC G-QL, Spearman’s rho 0.409–0.514, *P* < .0001). The FACIT-Sp-12 and NIH-HEALS each used other independent validated instruments for validity correlations (Spearman correlations between FACIT-Sp-12 and FACT-G quality of life, 0.09–0.62, Inverse correlation to POMS-SF, −0.21 to −0.60, and MCSDS, 0.22–0.27; NIH-HEALS correlation to FACIT-Sp-12 Meaning, Peace, and Faith factor scores, *r*_s_ = 0.60–0.62, *P* < 0.0001, and SIS Codependent factor inverse correlation, *r*_s_ = − 0.34, *P* < 0.0001).

Given substantial cultural changes in the way people relate to religion and spirituality in recent decades, with fewer claiming religious affiliation, it is important to evaluate the validity of spiritual wellbeing measures accordingly, especially in the context of psychedelics (Cherniak and Granqvist 2025). For example, items in the FACIT-Sp-12 are more vaguely worded, referencing “faith” and “spiritual beliefs,” rather than “God.” The NIH-HEALS and EORTC QLQ-SWB32 items move in the direction of spiritual practices, such as prayer and meditation, but also locate spirituality in relation to communities, relationships, or even the arts. Even when the EORTC QLQ-SWB32 does reference God, it provides non-theistic respondents the ability to skip items dealing with beliefs in God. These features of measuring spirituality and spiritual wellbeing are important in the context of PAT, as studies have suggested that the ways people hold and practice spiritual beliefs may change following interactions with psychedelic substances (Nayak et al. 2022; Griffiths et al. 2025). The more open-ended approach to spirituality may be among the reasons the FACIT-Sp-12 has been widely used in PAT studies.

In terms of patient-centered outcomes for palliative care patients, participant burden requires attention. The instruments with fewer items – the FACIT-Sp-12 (12), may be easier to complete alongside other outcome measures than the EORTC QLQ-SWB32 (32) and NIH-HEALS (35), especially considering the fatigue, pain, and other symptoms experienced by those with serious illness. However, the nuance provided by the longer questionnaires may also indicate unique aspects of the psychedelic experience not fully captured by shorter instruments. Stakeholder participation in the development is also an important patient-centered value to keep in mind here – the 3 measures each had patients, clinicians, and religious experts involved in item creation and selection, suggesting that values and needs of people with serious illness have been considered.

## Conclusion

As the re-emergence of PAT research is still in a nascent stage, there is a need for more standardization of research protocols and reporting (Pronovost-Morgan et al. 2025). This article asserts that spiritual wellbeing instruments should be used more frequently alongside descriptive questionnaires, such as the MEQ, to understand the implications of reported SERT experiences. Consistency in the use of outcome measures will assist researchers and clinicians in understanding mechanisms of action, the effects of medicines, as well as the therapeutic supports involved.

The use of an already well-regarded measure, like the FACIT-Sp-12, may represent the smoothest route to wider implementation. However, given the emergent dynamics of the field of psychedelic studies, the NIH-HEALS is also worthy of greater consideration for use in PAT. The fact that it has already been implemented in PAT studies (unlike the EORTC QLQ-SWB32) and that it has the institutional backing of a governmental agency may make this attractive to researchers pursuing FDA licensure of psychedelic medicines. Its strong psychometric properties, including validation in comparison to FACIT-Sp-12 domains (FACIT-Sp-12 Meaning, Peace, and Faith factor scores (*r*_s_ = 0.60–0.62, *P* < 0.0001), make it a credible measure. Though the NIH-HEALS is lengthy by comparison to FACIT-Sp-12, the items are inclusive of both spiritual beliefs and practices, relational connections, and even sources of support such as medical caregivers, giving this instrument the ability to identify various nuanced aspects of spiritual wellbeing in the face of serious illness make it compelling for use in PAT. Finally, the conceptual roots of the measure as a way of noting positive change in spiritual wellbeing in the face of serious illness make it compelling for use in PAT. Inasmuch as psychedelic medicines are interventions aimed at improving mental health conditions like depression, anxiety, demoralization, and PTSD, the NIH-HEALS may be uniquely suited to identify positive spiritual and emotional change along with other mental health outcomes.

The NIH-HEALS does have potential limitations that deserve further exploration in research. Given the positive change orientation of the instrument’s development, researchers will need to exercise caution in assessing the potential for introducing priming effects of the questionnaire itself on PAT outcomes. Having a positively oriented measure may risk failing to identify potentially negative spiritual outcomes from PAT, which have also been documented (Palitsky et al. 2024a, 2024b).

The NIH-HEALS measure is highly compatible with and could be used alongside the chaplain-led spiritual assessment interview discussed in the introduction. Future explorations are also warranted to evaluate spiritual wellbeing alongside other mental health outcomes in PAT, and to investigate the impact of SERT support efforts.

## References

[ref1] Aday JS, Mitzkovitz CM, Bloesch EK, et al. (2020) Long-term effects of psychedelic drugs: A systematic review. *Neuroscience and Biobehavioral Reviews* 113, 179–189. doi:10.1016/j.neubiorev.2020.03.017 32194129

[ref2] Addington-Hall J (2002) Research sensitivities to palliative care patients. *European Journal of Cancer Care* 11(3), 220–224. doi:10.1046/j.1365-2354.2002.00343.x 12296842

[ref3] Agin-Liebes GI, Malone T, Yalch MM, et al. (2020) Long-term follow-up of psilocybin-assisted psychotherapy for psychiatric and existential distress in patients with life-threatening cancer. *Journal of Psychopharmacology* 34(2), 155–166. doi:10.1177/0269881119897615 31916890

[ref4] Ameli R, Sinaii N, Luna MJ, et al. (2018) NIH-HEALS, a measure of psycho-social-spiritual healing, and its relationship to resilience and mindfulness. *Journal of Pain and Symptom Management* 56(6), e133. doi:10.1016/j.jpainsymman.2018.10.425

[ref5] Baker KM, Ulrich CM and Meghani SH (2023) An integrative review of measures of spirituality in experimental studies of psilocybin in serious illness populations. *American Journal of Hospice & Palliative Medicine* 40(11), 1261. doi:10.1177/10499091221147700 36604312

[ref6] Barrett FS, Johnson MW and Griffiths RR (2015) Validation of the revised mystical experience questionnaire in experimental sessions with psilocybin. *Journal of Psychopharmacology (Oxford, England)* 29(11), 1182–1190. doi:10.1177/0269881115609019 26442957 PMC5203697

[ref7] Beaussant Y (2023) *Pilot study of psilocybin-assisted therapy for demoralization in patients receiving hospice care - PATH study* (Clinical trial registration No. NCT04950608). clinicaltrials.gov. https://clinicaltrials.gov/study/NCT04950608(accessed 27 November 2025)

[ref8] Best MC, Simpson G, Jones KF, et al. (2024) Measurement of spiritual wellbeing in an australian hospital population using the functional assessment of chronic illness therapy: Spiritual wellbeing scale (FACIT-Sp-12). *Journal of Religion and Health* 63(5), 3714–3728. doi:10.1007/s10943-024-02064-x 38869732 PMC11502567

[ref9] Carpenter JG, Ulrich C, Hodgson N, et al. (2021) Alternative consent models in pragmatic palliative care clinical trials. *Journal of Pain and Symptom Management* 62(1), 183–191. doi:10.1016/j.jpainsymman.2020.09.044 33129936 PMC8108441

[ref10] Cherniak AD and Granqvist P (2025) How does psychedelic use relate to aspects of religiosity/spirituality? Preregistered report from a birth cohort study and a prospective longitudinal study. *Psychology of Religion and Spirituality* 17, 175–194. doi:10.1037/rel0000561

[ref11] Davis K, Palitsky R, Dunlop BW, et al. (2025) Psychedelic ethics in palliative care. *The American Journal of Bioethics* 25(1), 95–98. doi:10.1080/15265161.2024.2433457 39804326

[ref12] DeVon HA, Block ME, Moyle-Wright P, et al. (2007) A psychometric toolbox for testing validity and reliability. *Journal of Nursing Scholarship* 39(2), 155–164. doi:10.1111/j.1547-5069.2007.00161.x 17535316

[ref13] Fava M, Sorg E, Jacobs JM, et al. (2023) Distinguishing and treating demoralization syndrome in cancer: A review. *General Hospital Psychiatry* 85, 185–190. doi:10.1016/j.genhosppsych.2023.10.004 37950966

[ref14] Feng Y, Luo J, Lin T, et al. (2025) Translation and validation of the Chinese version of EORTC QLQ-SWB32 assessing the spiritual wellbeing of women with gynecological cancer. *PLOS ONE* 20(4), e0321790. doi:10.1371/journal.pone.0321790 40233117 PMC11999153

[ref15] Ferrell BR, Twaddle ML, Melnick A, et al. (2018) National consensus project clinical practice guidelines for quality palliative care guidelines, 4th Edition. *Journal of Palliative Medicine* 21(12), 1684–1689. doi:10.1089/jpm.2018.0431 30179523

[ref16] Galchutt PK (2024) Spiritual assessment models for palliative care chaplains: A narrative review. *Journal of Health Care Chaplaincy* 30(4), 329–345. doi:10.1080/08854726.2024.2368999 38900925

[ref17] Goyarrola R, Lipsanen J, Saarelainen S-M, et al. (2023) Spiritual well-being correlates with quality of life of both cancer and non-cancer patients in palliative care - further validation of EORTC QLQ-SWB32 in Finnish. *BMC Palliative Care* 22(1), 33. doi:10.1186/s12904-023-01153-0 36991431 PMC10061907

[ref18] Granda-Cameron C, Viola SR, Lynch MP, et al. (2008) Measuring patient-oriented outcomes in palliative care: Functionality and quality of life. *Clinical Journal of Oncology Nursing* 12(1), 65–77. doi:10.1188/08.CJON.65-77 18258576

[ref19] Griffiths RR, Jesse R, Richards WA, et al. (2025) Effects of psilocybin on religious and spiritual attitudes and behaviors in clergy from various major world religions. *Psychedelic Medicine* 3, 172–191. doi:10.1089/psymed.2023.0044 41869007 PMC13000417

[ref20] Griffiths RR, Johnson MW, Carducci MA, et al. (2016) Psilocybin produces substantial and sustained decreases in depression and anxiety in patients with life-threatening cancer: A randomized double-blind trial. *Journal of Psychopharmacology* 30(12), 1181–1197. doi:10.1177/0269881116675513 27909165 PMC5367557

[ref21] Griffiths RR, Richards WA, McCann U, et al. (2006) Psilocybin can occasion mystical-type experiences having substantial and sustained personal meaning and spiritual significance. *Psychopharmacology* 187(3), 268–283. doi:10.1007/s00213-006-0457-5 16826400

[ref22] Grob CS (2025) *Pragmatic trial of psilocybin therapy in palliative care (PT2PC): a multicenter triple-blind phase 2 randomized controlled trial of psilocybin therapy for demoralized adults near the end of life* (Clinical trial registration No. NCT05403086). clinicaltrials.gov. https://clinicaltrials.gov/study/NCT05403086 (accessed 27 November 2025)

[ref23] Holder GN, Young WC, Nadarajah SR, et al. (2015) Psychosocial experiences in the context of life-threatening illness: The cardiac rehabilitation patient. *Palliative and Supportive Care* 13(3), 749–756. doi:10.1017/S1478951514000583 24892820

[ref24] Ko K, Knight G, Rucker JJ, et al. (2022) Psychedelics, mystical experience, and therapeutic efficacy: A systematic review. *Frontiers in Psychiatry* 13. https://www.frontiersin.org/articles/10.3389/fpsyt.2022.917199 (accessed 13 August 2023) 10.3389/fpsyt.2022.917199 PMC9340494 35923458

[ref25] Kyranou M and Nicolaou M (2021) Associations between the spiritual well-being (EORTC QLQ-SWB32) and quality of life (EORTC QLQ-C30) of patients receiving palliative care for cancer in Cyprus. *BMC Palliative Care* 20(1), 133. doi:10.1186/s12904-021-00830-2 34461881 PMC8404401

[ref26] Marks M, Brendel RW, Shachar C, et al. (2024) Essentials of informed consent to psychedelic medicine. *JAMA Psychiatry* 81, 611. doi:10.1001/jamapsychiatry.2024.0184 38598209

[ref27] Marks M and Cohen IG (2023) How should the FDA evaluate psychedelic medicine? *The New England Journal of Medicine* 389(19), 1733–1735. doi:10.1056/NEJMp2308940 37929835

[ref28] McCann Klug L (2023) The specialty chaplain on the palliative care team: A narrative review. *American Journal of Hospice and Palliative Medicine®* 40(9), 1021–1028. doi:10.1177/10499091221134021 36226868

[ref29] Miller M, Rosa WE, Doerner Rinaldi A, et al. (2023) Applying key lessons from the hospice and palliative care movement to inform psychedelic-assisted therapy. *Psychedelic Medicine* 1(3), 124–129. doi:10.1089/psymed.2022.0009 37753521 PMC10518906

[ref30] Murphy R, Kettner H, Zeifman R, et al. (2021) Therapeutic alliance and rapport modulate responses to psilocybin assisted therapy for depression. *Frontiers in Pharmacology* 12, 788155. doi:10.3389/fphar.2021.788155 35431912 PMC9009076

[ref31] Nayak S, Singh M, Yaden DB, et al. (2022, June 30) Belief changes associated with psychedelic use. PsyArXiv. doi:10.31234/osf.io/rdzmy. 36317643

[ref32] Palitsky R, Canby NK, Van Dam NT, et al. (2024a) Leveraging meditation research for the study of psychedelic-related adverse effects. *International Review of Psychiatry* 36(8), 841. doi:10.1080/09540261.2024.2420745 39980218

[ref33] Palitsky R, Kaplan DM, Peacock C, et al. (2023) Importance of integrating spiritual, existential, religious, and theological components in psychedelic-assisted therapies. *JAMA Psychiatry* 80, 743. doi:10.1001/jamapsychiatry.2023.1554 37256584

[ref34] Palitsky R, Kaplan DM, Perna J, et al. (2024b) A framework for assessment of adverse events occurring in psychedelic-assisted therapies. *Journal of Psychopharmacology* 38, 690–700. doi:10.1177/02698811241265756 39082259

[ref35] Pasricha I, Peacock C, Palitsky R, et al. (2025) What motivates spiritual health practitioners in psychedelic-assisted therapy? A qualitative study and implications for facilitator training practices. *Psychedelics* 1, 31–39. doi:10.61373/pp025r.0008

[ref36] Peacock C, Mascaro JS, Brauer E, et al. (2024) Spiritual health practitioners’ contributions to psychedelic assisted therapy: A qualitative analysis. *PLOS ONE* 19(1), e0296071. doi:10.1371/journal.pone.0296071 38166057 PMC10760908

[ref37] Peterman AH, Fitchett G, Brady MJ, et al. (2002) Measuring spiritual well-being in people with cancer: The functional assessment of chronic illness therapy—spiritual well-being scale (FACIT-Sp). *Annals of Behavioral Medicine* 24(1), 49–58. doi:10.1207/S15324796ABM2401_06 12008794

[ref38] Peterman AH, Reeve CL, Winford EC, et al. (2014) Measuring meaning and peace with the FACIT–spiritual well-being scale: Distinction without a difference? *Psychological Assessment* 26(1), 127–137. doi:10.1037/a0034805 24188147 PMC4081471

[ref39] Pronovost-Morgan C, Greenway KT and Roseman L (2025) An international Delphi consensus for reporting of setting in psychedelic clinical trials. *Nature Medicine* 31, 1–10. doi:10.1038/s41591-025-03685-9 PMC12283393 40461819

[ref40] Ross S, Agrawal M, Griffiths RR, et al. (2022) Psychedelic-assisted psychotherapy to treat psychiatric and existential distress in life-threatening medical illnesses and palliative care. *Neuropharmacology* 216, 109174. doi:10.1016/j.neuropharm.2022.109174 35772523

[ref41] Ross S, Bossis A, Guss J, et al. (2016) Rapid and sustained symptom reduction following psilocybin treatment for anxiety and depression in patients with life-threatening cancer: A randomized controlled trial. *Journal of Psychopharmacology* 30(12), 1165–1180. doi:10.1177/0269881116675512 27909164 PMC5367551

[ref42] Shnayder S, Ameli R, Sinaii N, et al. (2023) Psilocybin-assisted therapy improves psycho-social-spiritual well-being in cancer patients. *Journal of Affective Disorders* 323, 592–597. doi:10.1016/j.jad.2022.11.046 36513161 PMC9884542

[ref43] Sloan DH, BrintzenhofeSzoc K, Kichline T, et al. (2017) An assessment of meaning in life-threatening illness: Development of the Healing Experience in All Life Stressors (HEALS). *Patient Related Outcome Measures* 8, 15–21. doi:10.2147/PROM.S118696 28243158 PMC5317311

[ref44] Vivat B, Young T, Efficace F, et al. and on behalf of the EORTC Quality of Life Group (2013) Cross-cultural development of the EORTC QLQ-SWB36: A stand-alone measure of spiritual wellbeing for palliative care patients with cancer. *Palliative Medicine* 27(5), 457–469. doi:10.1177/0269216312451950 22843128

[ref45] Vivat B, Young TE, Winstanley J, et al. and Group O behalf of the EQ of L (2017) The international phase 4 validation study of the EORTC QLQ-SWB32: A stand-alone measure of spiritual well-being for people receiving palliative care for cancer. *European Journal of Cancer Care* 26(6), e12697. doi:10.1111/ecc.12697 28776784

[ref46] Wheelwright S, Bjordal K, Bottomley A, et al. (2021) EORTC quality of life group guidelines for developing questionnaire modules. https://qol.eortc.org/manual/the-module-development-guidelines-2021/ (accessed 28 June 2025)

[ref47] Whinkin E, Eparwa TRJ, Julseth MC, et al. (2023) Reductions in anxiety and depression symptoms in a subset of outpatients with problematic substance use who received ketamine-assisted psychotherapy: A two-year retrospective chart review. *Frontiers in Psychiatry* 14, 1160442. doi:10.3389/fpsyt.2023.1160442 37711421 PMC10498542

[ref48] Yaden DB and Griffiths RR (2021) The subjective effects of psychedelics are necessary for their enduring therapeutic effects. *ACS Pharmacology & Translational Science* 4(2), 568–572. doi:10.1021/acsptsci.0c00194 33861219 PMC8033615

[ref49] Zarrabi A (2023) The safety, feasibility, and acceptability of psilocybin combined with multidisciplinary palliative care in demoralized cancer survivors with chronic pain (P-PC) (Clinical trial registration No. NCT05506982). clinicaltrials.gov. https://clinicaltrials.gov/study/NCT05506982 (accessed 27 November 2025)

